# Finite Element Modeling of Laminated Composite Plates with Locally Delaminated Interface Subjected to Impact Loading

**DOI:** 10.1155/2014/954070

**Published:** 2014-02-13

**Authors:** Saddam Hussein Abo Sabah, Ahmad Beng Hong Kueh

**Affiliations:** Construction Research Centre, Universiti Teknologi Malaysia (UTM-CRC), 81310 Johor Bahru, Johor, Malaysia

## Abstract

This paper investigates the effects of localized interface progressive delamination on the behavior of two-layer laminated composite plates when subjected to low velocity impact loading for various fiber orientations. By means of finite element approach, the laminae stiffnesses are constructed independently from their interface, where a well-defined virtually zero-thickness interface element is discreetly adopted for delamination simulation. The present model has the advantage of simulating a localized interfacial condition at arbitrary locations, for various degeneration areas and intensities, under the influence of numerous boundary conditions since the interfacial description is expressed discretely. In comparison, the model shows good agreement with existing results from the literature when modeled in a perfectly bonded state. It is found that as the local delamination area increases, so does the magnitude of the maximum displacement history. Also, as top and bottom fiber orientations deviation increases, both central deflection and energy absorption increase although the relative maximum displacement correspondingly decreases when in contrast to the laminates perfectly bonded state.

## 1. Introduction

Interfacial imperfection that eventually leads to the delamination of laminated composite plates is commonly considered as one of the chief contributors to performance degradation of these advanced lightweight materials. This imperfection is structurally harmful and contributes principally to the failure of the laminate especially in presence of dynamic loading environment such as impact loading. The imperfection in the interface may be due to insufficient adhesion or incorrect equipment setting during manufacturing, leading it to undergo serious damage such as matrix cracks, fibers breakages, or delamination. These kinds of damage are severe because they drastically reduce the mechanical characteristics of the laminate and at the same time can leave visible marks on the impacted surface. Therefore, understanding the interface condition effect on laminated composite plates is of great importance for improvement of the plate's strength.

In terms of loading environment, laminated composite plates are prone to impact loads that occur during manufacturing, transportation, or service life. These impact loads are considered extremely dangerous and cause invisible damage to the back face or within the laminate which consequently reduce the strength of the composite material, even when the impact produces low energy. It has been proven that if a composite plate is subjected to a low velocity impact, the damaged area will increase with the increase of the impact velocity [1]. Caprino et al. [2] concluded that the first failure point in a laminated composite plate is influenced more by the impact speed rather than the impacting energy. In much similar vein, a finite element model was developed to study the response of laminated composite folded plates subjected to impact loads [3]. The results indicate that, under impact loading, the amplitude and period of a laminated composite folded plate tend to increase. The damage caused by the impact load is very obvious on the face of the plate.

In general, there exist several factors that significantly influence the behavior of composite materials when subjected to impact loading. They can basically be divided into two parts. The first part involves the laminate properties such as shape, size, thickness, ply orientation, and stacking sequence. The second category corresponds to impactor properties such as impactor shape, velocity, and energy.

In regard to the thickness effect of the composite, an extensive study on thin and standard CFRP laminates was performed by Shi et al. [4] to observe the impact damage mechanism, from which it was found that the thin laminate showed a higher strength than the standard laminate by 23% difference. The transverse cracks were lower in the thin-ply laminate, although the delamination area was larger. Furthermore, the compression after impact strength of the thin laminate was also higher by 23%. Chen et al. [5] studied extensively the thickness effect on the contact behavior of a laminated composite plate indented by a rigid sphere. They found that the thickness has a negligible effect if the plate thickness is greater than 2 mm. However, the thickness of the composite plate has a real significant effect on the force-indentation response, if the plate thickness is less than 2 mm. It is worth mentioning that the delamination area also depends on the laminate thickness. A low velocity impact test on graphite fabric/epoxy laminates having different thicknesses was performed by Caprino et al. [2]. It was found that the energy at the onset of delamination is shared in two parts. The first part belongs to the specimen flexural deformation and the second to the deformation at the point of contact which depends on the laminate thickness. Aboussaleh and Boukhili [6] had demonstrated that the resulting indentation from the impact increases as the plate thickness increases. Three laminated plates of carbon-epoxy with different thicknesses of 4, 8, and 16 layers were tested by Belingardi and Vadori [7] to study the thickness effect on the global impact behavior of the laminate. The plates were stacked in two different staking sequences: [0/90]_*i*_ and [0/60/−60]_*i*_. It was observed that the saturation energy increases as the laminate thickness increases.

Tiberkak et al. [8] studied the effects of the stacking sequence on fiber-reinforced composite plates subjected to low velocity impact. The results show that increasing the 90° plies reduces the rigidity of the laminate. Will et al. [9] claimed that the stacking sequence of the laminate affects the total delamination area, the delamination location, and the shear fracture area. The impact response of continuous fiber-reinforced composites with different stacking sequences was also studied by Cantwell and Morton [10]. It was concluded that the fiber stacking sequence determines both the elastic energy absorbing capability and the failure mode of the composite. They recommended that the abruption of fiber orientation such as ±45° interfaces should be avoided especially under low velocity impacts.

Many researches have been conducted regarding how the fiber orientation influences the impact behavior of laminated composites. The effect of laminate reinforcement arrangement on the behavior of polymer matrix composites was studied using a simply supported plate with dimensions of 150 mm × 150 mm × 6 mm [11]. It was noticed that when mixing unidirectional and woven-fabric layers, the overall failure is dramatically decreased when subjected to impact load, suggesting that mixed composites are more resistant to impact damage. A study on the effect of plate geometry on the crushing of flat composite plates was conducted by Cauchi Savona and Hogg [12]. It was found that the specific crushing stresses are significantly influenced by the fiber type and arrangement. The quadriaxial oriented laminates showed better crushing efficiencies and consistency than the triaxial orientations, due to higher amount of fibers with 0° orientation in the triaxial laminates which makes the crushing stress require long strokes in order to be stable. They also noticed that if the outer plies delaminate without any damage, the specific crushing stresses will automatically decrease. The effect of both fiber orientation and stacking sequence on composites using unidirectional (UD) and multidirectional (MD) composite laminates was studied by Naghipour et al. [13]. It was demonstrated that the load-displacement response of MD composites relies mainly on the stacking sequence of the laminate. The damage tolerance of polyester laminates subjected to low velocity impact at inclined angles using a hemispherical-ended cylindrical steel impactor was studied by Madjidi et al. [14]. They reported that increasing the inclination angle leads to a reduction in the damage area and the depth of surface indentation.

The low velocity impact of E-glass/epoxy laminated composite plates using three rectangular plates of 150 mm × 50 mm, 150 mm × 100 mm, and 150 mm × 150 mm dimensions was studied by Aslan et al. [15]. It was noticed that the shape of the force changes as the mass varies. The plate of 150 mm × 100 mm dimensions experienced the maximum impact force compared to the other two. The area of delamination of this plate was wider as well, implying that the smaller the width of the laminate, the higher the contact duration. Also, Robinson and Davies [16] reported that the increase in the size of laminated composites that are prone to low velocity impact leads to an increase in the impact energy that causes severe damage. Therefore, it was suggested that a damage energy approach should be used to help predict the impact energy in a differently sized laminated composite.

The effects of high velocity impacts (ballistic impact) on carbon/epoxy composite panels using hemispherical, conical, fragment simulating, and flat impactors were studied by Ulven et al. [17]. It was observed that, under high velocity impact, the conical impactor causes the largest amount of absorbed energy. This is followed by the flat impactor, the hemispherical impactor, and then the fragment simulating impactor. Three different impactors: hemispherical, conic with 30°, and conic with 90°, were used to study the response of fiber polyester composite laminates by Dhakal et al. [18] employing tests that were performed at four different velocities: 2.52 m/s, 2.71 m/s, 2.89 m/s, and 2.97 m/s. The composite laminates that were impacted by the hemispherical impactor had the ability to take the maximum load. For this reason, the hemispherical impactor caused the largest damaged area on both faces of the specimens. The damage on the back face of the laminate was severe for all the three impactors compared to the front face damage and the back damage increases with the increase in the velocity. The ability of the specimens tested with the 90° impactor to resist damage was higher than the ones tested with the 30° impactor. The contribution of impactor shape to resulting damage and response of composite laminates was investigated using carbon-fiber/epoxy laminated plates subjected to low velocity by Mitrevski et al. [19]. Three different types of impactors including steel hemispherical, ogival, and conical with the same diameter of 12 mm were considered. They found that the absorbing energy of the carbon-fiber/epoxy laminated plate for the conical impactor was the highest. The conical impactor also produced the largest penetration depth. For the hemispherical impactor, the peak force was the greatest and the force contact duration was the shortest. In terms of damage threshold load, the hemispherical impactor achieved the highest followed by those of the ogival and conical impactors, respectively.

In connection with the interfacial degeneration behaviors, early works on the imperfect bonding had been focused on the shear slip in cross-ply laminates adopting Pagano's analytical solutions [20–22], due to limited experimental investigation. Toledano and Murakami [23] considered the shear slip in a two-layer cross-ply composite laminate (length-to-thickness ratio, *S* = 6) through the inclusion of both linear and nonlinear interface slip laws, which had been proven to be valid in analyzing the beam with an interlayer slip [24]. The bending response of a laminate with the same stacking sequence had been examined by Lu and Liu [25] using the Interlayer Shear Slip Theory (ISST) and others [26–29] using the linear spring-layer model, in which the mid-plane deflection under a variety of shear slip coefficients as well as through-thickness mid-point deflection was addressed. da Silva and Sousa [30] presented a family of interface elements employing the Euler-Bernoulli and Timoshenko beam theories for the analysis of composite beams with an interlayer slip, from which the former was claimed preferable for simplicity, whereas the latter had been shown to produce the most accurate structural responses, free of spurious slip strain distribution and shear locking even when high connection stiffness was considered. In addition to shear slip, the weak bonding modeling which includes a normal opening had been investigated by Shu and Soldatos [28], Soldatos and Shu [31], Williams [32], and Williams and Addessio [33] considering two-layer cross-ply laminates where their effects on the through-thickness mid-point deflection were studied. In this coupled condition, the thickness of laminate in relation to its surface dimensions plays a dominant role. Taking into account this particular parameter, the sensitivity of the plate, with different length-to-thickness ratios, to a complete debonding was explored in terms of mid-point deflection in Williams [32] and Williams and Addessio [33]. Moreover, the influence of different extents of bonding, ranging from a perfect bonding to complete debonding, on the mid-point bending response of laminates with *S* = 4, 10, and 100 had been highlighted in Soldatos and Shu [31]. Such an effect had also been examined in two-layer laminates with a symmetric lay-up [28, 31, 34–36] and an antisymmetric lay-up [37]. With regard to the symmetric laminate, Liu et al. [35, 36] and Soldatos and Shu [31] had reported the mid-point bending response of plate under various combinations of axial and normal imperfection, whereas a uniform degradation had been assumed in both directions in Soldatos and Shu [31]. From the standpoint of fiber orientation, Kam et al. [38] studied interfacial degeneration effects on the bending response of two-layer laminates, in which a generalization polar plot that incorporates numerous affecting parameters was constructed.

Although numerous studies were devoted to impact behavior of composite plates, it should be noted that there exists little literature evidence on the effects of delaminated interface in particular that exists locally. The current paper aims to fill this gap by focusing on the behavior of two-layer laminates subjected to low velocity impact. The paper is arranged as follows. After the introduction, we present the finite element modeling procedure for a two-layer composite plate, consisting of two laminae with a well-defined virtually zero-thickness interface element in between. The effects of locally delaminated laminates which vary in area from the center outwards are then discussed in the presence of impact loading. In the end, we conclude the main findings of our study.

## 2. Numerical Procedure 

### 2.1. Model Description

**Figure 1:**
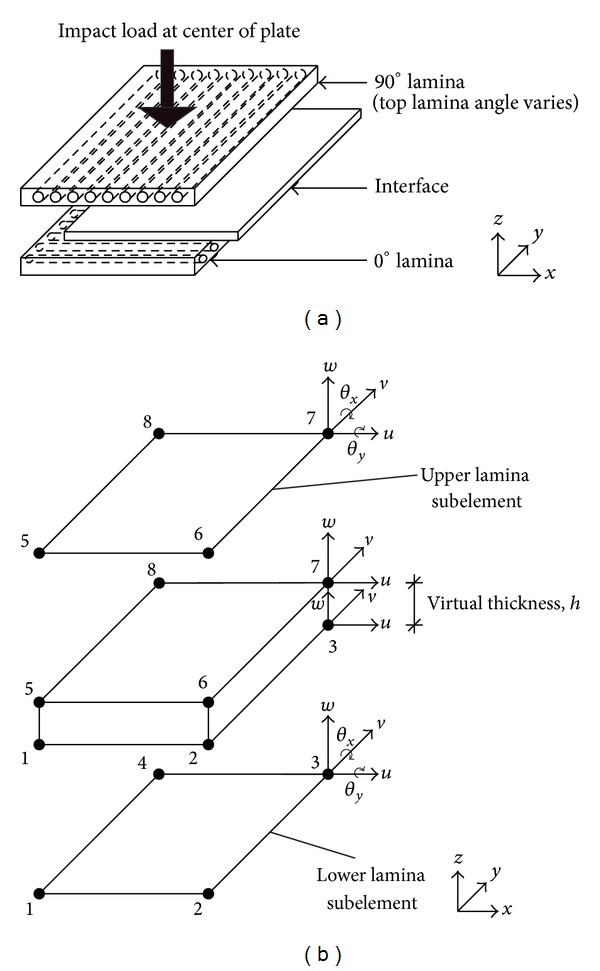
(a) Configuration of composite laminate. (b) DOF of lamina subelements and an interface element that lies in between.

Figure 1 shows the finite element model (FEM) used in this study. The bottom layer has a fixed 0° fiber orientation, while that of the upper layer changes from 0° up to 90°. The study is restricted to a rectangular laminate plate, which is considered to be thin and flat. It consists of two layers of lamina with equal thickness and an interface layer between them. Each lamina is formed by unidirectional fibers of E-glass and the matrix material is epoxy (3501-6). The lamina is considered as a transversely isotropic solid material. The dimensions of the plate are 100 mm × 100 mm with a total thickness of 0.5 mm. The initial velocity of the impactor is 1 m/s having a 0.1 kg weight and 0.002 s impact duration. This time span is chosen such that an appreciable deformation can be observed in simulation. Only impacts of low velocity are considered in this study. The boundary condition of the plate is fully clamped at all edges. In addition, the load is applied at the center of the plate without taking into consideration the impactor shape. It is worth mentioning that damping is not considered in this study. It should be noted that the influence of damping is somewhat practically negligible in most structural applications unless specialized treatment is offered in the material fabrication. Moreover, its effect is not the chief concern of our current investigation. To model the imperfect interfacial effect, we simulate the progressive delamination, which spreads outward from the center of laminate to its edges at the interfacial region.

### 2.2. Numerical Model Construction

The governing equation of this study can be formulated as follows:

(1) $$\begin{array}{c} \left[M\right]\left\{\ddot{u}\right\}+\left[K\right]\left\{u\right\}=\left\{F\right\}, \end{array}$$

where *M* is the global mass matrix of the laminate, *K* is the global stiffness matrix of the laminate, and interface *F*, *u*, and $\ddot{u}$ are the impact force vector matrix, displacement, and acceleration, respectively.

#### 2.2.1. Computation of Laminate Stiffness Matrix

The stiffness of the laminate is computed by adding up the stiffness of each subelement in all layers in the laminate including the interface layer. The elemental stiffness matrix is computed by relating the *ABD* matrix of the lamina to the element strain matrix as shown in

(2) K=∬[BiT(A)ABDBi+BiT(B)ABDBo+BoT(B)ABDBi+BoT(D)ABDBo]|J|dζ dη,

where

(3) $$\begin{array}{c} J=\left[\begin{array}{cc} \frac{\partial x}{\partial \xi } & \frac{\partial y}{\partial \xi }\\ \frac{\partial x}{\partial \eta } & \frac{\partial y}{\partial \eta } \end{array}\right], \end{array}$$

*K* is the lamina subelement stiffness matrix [20 × 20], *B*_*i*_ is the in-plane strain-displacement matrix [3 × 8], *B*_*o*_ is the out-of-plane strain-displacement matrix [3 × 12], (*A*)_*ABD*_ is the extensional stiffness [3 × 3] of the lamina, (*B*)_*ABD*_ is the coupling stiffness [3 × 3] of the lamina, (*D*)_*ABD*_ is the bending stiffness [3 × 3] of the lamina, and *J* is the Jacobian matrix.

In terms of the FEM description, each lamina is modeled by a four-node lamina subelement. The corresponding arrangements of nodes and degrees of freedom (DOF) are shown in Figure 1(b). The numbering of nodes in the lamina subelement is arranged in anticlockwise manner and each node has 5 DOF which include displacements in *x*-, *y*-, and *z*-directions (*u*, *v*, and *w*, resp.) as well as rotations about *y*- and *x*-directions (*θ*_*x*_ and *θ*_*y*_, resp.). The displacements of lamina subelement are expressible as

(4) $$\begin{array}{c} u=\left[N_{i}\right]\left\{u_{i}\right\};\;\;v=\left[N_{i}\right]\left\{v_{i}\right\};\;\;w=\left[N_{o}\right]\left\{w_{i}\right\}, \end{array}$$

where *N*_*i*_ and *N*_*o*_ are, respectively, the in-plane Lagrange shape function and out-of-plane polynomial shape function of a nonconforming rectangular element with 12 terms. In detail, {*u*_*i*_} = {*u*_1_ *u*_2_ *u*_3_ *u*_4_}^*T*^, {*v*_*i*_} = {*v*_1_ *v*_2_ *v*_3_ *v*_4_}^*T*^, and {*w*_*i*_} = {*w*_1_ *θ*_1*x*_ *θ*_1*y*_ *w*_2_ *θ*_2*x*_ *θ*_2*y*_ *w*_3_ *θ*_3*x*_ *θ*_3*y*_ *w*_4_ *θ*_4*x*_ *θ*_4*y*_}^*T*^.

The stiffness matrices of the lamina subelements are assembled in the local stiffness matrix of the laminate element as follows:

(5) $$\begin{array}{c} K_{\text{LAM}}=\left[\begin{array}{cc} K_{\text{lower}} & K_{\text{null}}\\ K_{\text{null}} & K_{\text{upper}} \end{array}\right], \end{array}$$

where *K*_LAM_ is the stiffness matrix [40 × 40] of laminate element, *K*_lower_ is the stiffness matrix [20 × 20] of bottom lamina subelement, *K*_null_ is the null matrix [20 × 20], and *K*_upper_ is the stiffness matrix [20 × 20] of top lamina subelement.

#### 2.2.2. Stiffness Matrix of Interface

For this study, we adopt here for the interface layer a virtually zero-thickness interface element. There are eight nodes in the zero-thickness interface element, the node sequence of which is arranged in anticlockwise manner from bottom to top as shown in Figure 1(b). Each node in the zero-thickness interface element contains 3 DOF represented by the Lagrange shape functions in

(6) $$\begin{array}{c} \left\{d_{\text{bot}}\right\}=\left[N\right]\left\{\bar{d_{\text{bot}}}\right\};\;\;\left\{d_{\text{top}}\right\}=\left[N\right]\left\{\bar{d_{\text{top}}}\right\}, \end{array}$$

where {*d*_bot_} = {*u*_bot_ *v*_bot_ *w*_bot_}^*T*^,{*d*_top_} = {*u*_top_ *v*_top_ *w*_top_}^*T*^, $\left\{\bar{d_{\text{bot}}}\right\}={\left\{u_{1}\;v_{1}\;w_{1}\;u_{2}\;v_{2}\;w_{2}\;u_{3}\;v_{3}\;w_{3}\;u_{4}\;v_{4}\;w_{4}\right\}}^{T}$, {*d*_top_} = {*u*_5_ *v*_5_ *w*_5_ *u*_6_ *v*_6_ *w*_6_ *u*_7_ *v*_7_ *w*_7_ *u*_8_ *v*_8_ *w*_8_}^*T*^, {*d*_bot_} is the interpolated displacement [3 × 1] of the node at lower surface of the zero-thickness element, {*d*_top_} is the interpolated displacement [3 × 1] of the node at upper surface of the zero-thickness element, [*N*] is the Lagrange shape function [3 × 12], and $\left\{\bar{d_{\text{bot}}}\right\}$ and $\left\{\bar{d_{\text{top}}}\right\}$ are the nodal numbers of the interface element.

It should be noted that the shape function of the zero-thickness interface element in this study is a 2-dimensional Lagrange shape function rather than that of a 3-dimensional one although the interface element resembles the geometrical configuration of a solid element. Since the interface in this study is considered an orthotropic material with zero normal stresses in *x*- and *y*-directions (*σ*_*x*_ = 0 and *σ*_*y*_ = 0) and zero in-plane shear stress in *x*-*y* plane (*τ*_*xy*_ = 0), the stress-strain relationship of the zero-thickness element can be stated as

(7) $$\begin{array}{c} \left\{\sigma \right\}=\left[D_{\mathrm{int}}\right]\left\{\varepsilon \right\}, \end{array}$$

where

(8) $$\begin{array}{c} \left\{\sigma \right\}=\left\{\begin{array}{c} \tau_{xz}\\ \tau_{yz}\\ \sigma_{z} \end{array}\right\},\;\;\left\{\varepsilon \right\}=\left\{\begin{array}{c} \gamma_{xz}\\ \gamma_{yz}\\ \varepsilon_{z} \end{array}\right\}, \end{array}$$

and the constitutive matrix, [*D*_int⁡_], is given by

(9) $$\begin{array}{c} \left[D_{\mathrm{int}}\right]=\left[\begin{array}{ccc} G_{xz}\left(1-R\right) & 0 & 0\\ 0 & G_{yz}\left(1-R\right) & 0\\ 0 & 0 & E_{z}\left(1-R\right) \end{array}\right], \end{array}$$

where *G*_*xz*_ and *G*_*yz*_ are the in-plane shear moduli, *E*_*z*_ is Young's modulus in the *z*-direction, and *R* is the degeneration ratio that represents the extents of degeneration of interface; that is, 0 ≤ *R* ≤ 1 (fully delaminated).

In the present study, *R* = 1 is induced to model the case of delamination. We define further the delamination area ratio as follows:

(10) Area ratio  (Ar) =area of delamination for delaminated laminatearea of interface for perfectly bonded laminate.

In addition, the deformation in the zero-thickness element is computed from the relative displacement as follows:

(11) {ε}=1h{ΔuΔvΔw},

where {*ε*} is the deformation in the zero-thickness element, Δ*u* = *u*_upper_ − *u*_lower_, Δ*v* = *v*_upper_ − *v*_lower_, Δ*w* = *w*_upper_ − *w*_lower_, Δ*u*, Δ*v*, and Δ*w* are the relative displacement and *h* is the element thickness.

In this study, we only study the localized imperfection with a uniform degeneration in *G*_*xz*_, *G*_*yz*_, and *E*_*z*_, although different degradation can be imposed on one or more of these properties by using different values of *R* in [*D*_int⁡_]. From (6) and (11), the strains in the zero-thickness element are related to its nodal displacements via

(12) $$\begin{array}{c} \left\{\varepsilon \right\}=\left[B_{\mathrm{int}}\right]\left\{\overset{\wedge }{d}\right\}, \end{array}$$

where [*B*_int⁡_] = (1/*h*)[−*N* *N*], $\left\{\overset{\wedge }{d}\right\}={\left\{\bar{d_{\text{bot}}}\;\bar{d_{\text{top}}}\right\}}^{T}$, and [*B*_int⁡_] and $\left\{\overset{\wedge }{d}\right\}$ are the element strain-displacement matrix and nodal displacements of the zero-thickness element, respectively. Therefore, the elemental stiffness matrix of the zero-thickness interface is computed using the following equation:

(13) $$\begin{array}{c} K_{\mathrm{int}}=\frac{1}{h}\iint_{-1}^{1}B_{\mathrm{int}}^{T}D_{\mathrm{int}}B_{\mathrm{int}}\left|J\right|d\zeta \;d\eta . \end{array}$$

#### 2.2.3. Computation of Mass Matrix

For the mass matrix of the laminated composite plate, the consistent mass matrix is employed. The mass matrix [*M*] is the sum of the element mass matrix [*m*] as follows:

(14) $$\begin{array}{c} \left[M\right]=\overset{n}{\underset{e=1}{\sum }}\left[m\right], \end{array}$$

where *n* is the number of total elements.

The element mass submatrices are given as follows:

(15) $$\begin{array}{c} \left[m\right]=\int \overset{}{\underset{A}{\int }}{\rho hN}^{T}N\;dA, \end{array}$$

where *ρ* is the material mass density and *N* is the element shape function.

#### 2.2.4. Computation of Force Vector

Newmark's numerical time integration method is employed to calculate the time-dependent impact loading, which is applied to the plate. Newmark's integration scheme starts by assuming initial force (*F*_0_), displacement (*U*_0_), velocity $\left({\overset{`}{U}}_{0}\right)$, and acceleration $\left({\ddot{U}}_{0}\right)$ vectors. A time step (Δ*t*) is then chosen in order to calculate the impact loading exerted on the plate. For zero damping, Newmark's method is conditionally stable, if *γ* ≥ 0.5 and *β* ≤ 0.5, where *γ* and *β* are Newmark's method parameters. The method has basically eight integration constants and they are computed as follows:

(16) $$\begin{array}{c} a_{0}=\frac{1}{\beta \Delta t^{2}},\;\;a_{1}=\frac{\gamma }{\beta \Delta t},\\ a_{2}=\frac{1}{\beta \Delta t},\;\;a_{3}=\left(\frac{1}{2\beta }\right)-1,\\ a_{4}=\left(\frac{\gamma }{\beta }\right)-1,\;\;a_{5}=\left(\frac{\Delta t}{2}\right)\left(\left(\frac{\gamma }{\beta }\right)-2\right),\\ a_{6}=\Delta t\left(1-\gamma \right),\;\;a_{7}=\gamma \Delta t. \end{array}$$

For each time step, the effective load is calculated at time *t* + Δ*t* and it is given by

(17) $$\begin{array}{c} {\;}^{t+\Delta t}F_{\text{eff}}={\;}^{t+\Delta t}F+M\left({a_{0}}^{t}U+{a_{2}}^{t}\dot{U}+{a_{3}}^{t}\ddot{U}\right), \end{array}$$

where *F*_eff_ is the effective load, *F* is the force vector, and *U*,  $\dot{U}$,  and  $\ddot{U}$ are the displacement, velocity, and acceleration matrices, respectively.

The velocities and accelerations can also be computed by first computing the effective stiffness matrix:

(18) $$\begin{array}{c} K_{\text{eff}}=K+a_{0}M, \end{array}$$

where *K*_eff_  is the effective stiffness matrix and *K* is the lamina stiffness matrix.

Solving for displacement at time *t* + Δ*t*,

(19) $$\begin{array}{c} K_{\text{eff}}^{t+\Delta t}U={\;}^{t+\Delta t}F_{\text{eff}}. \end{array}$$

Thus, the accelerations and velocities are computed as, respectively,

(20) $$\begin{array}{c} {\;}^{t+\Delta t}\ddot{U}=a_{0}\left({\;}^{t+\Delta t}U-{\;}^{t}\dot{U}\right)-{a_{2}}^{t}\dot{U}-{a_{3}}^{t}\ddot{U}, \end{array}$$

(21) $$\begin{array}{c} {\;}^{t+\Delta t}\dot{U}={\;}^{t}\dot{U}+{a_{6}}^{t}\ddot{U}+{a_{7}}^{t+\Delta t}\ddot{U}. \end{array}$$

### 2.3. Verification of Model

The time step chosen for this study is 1.4 ms following the study of Shi et al. [4] on the vibration analysis of laminated plates for the purpose of validation. The same material properties and plate dimensions used in their study as shown in Table 1 are adopted for comparison.

Table 2 shows the natural frequencies of the first eight mode shapes obtained from the present study and those obtained from Shi et al. [4]. From the results, it can be seen that the maximum difference between the two natural frequencies is 5.45%. This difference may be due to the fact that Shi et al. [4] used the Galerkin approach to the partial differential equations through the Fourier series, while the present study uses the finite element method. Nonetheless, it is evident that current model offers close agreement to Shi et al. [4].

## 3. Results and Discussion

### 3.1. Central Displacement

**Figure 2:**
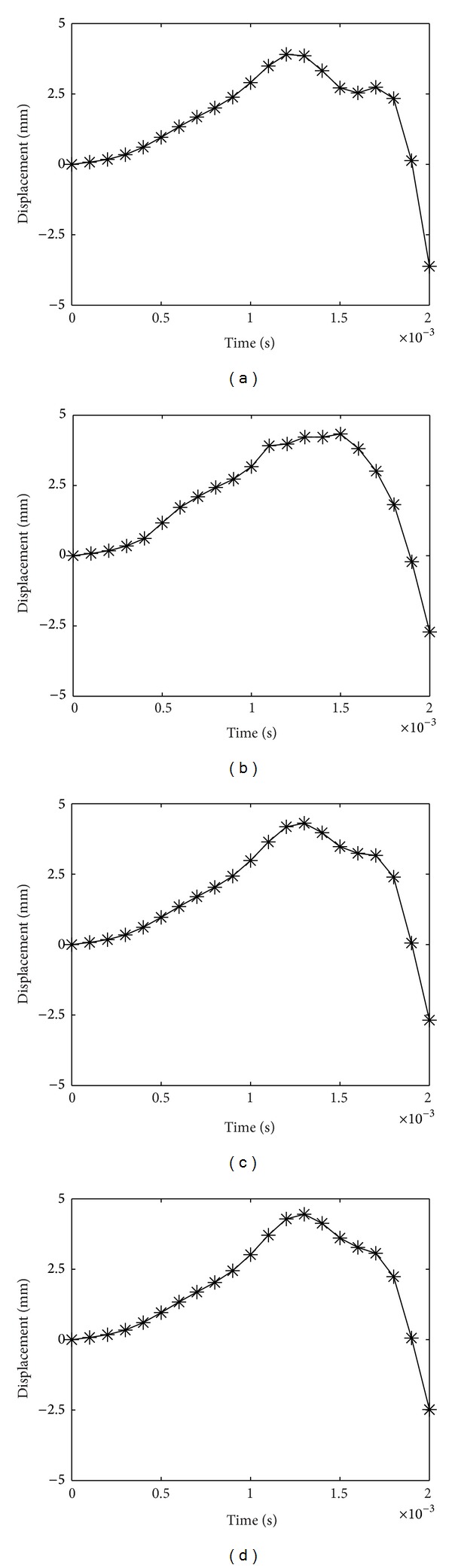
Displacement history of laminate with *A*_*r*_ = 0.25 for (a) [0/0], (b) [30/0], (c) [50/0], and (d) [90/0] laminates.

**Figure 3:**
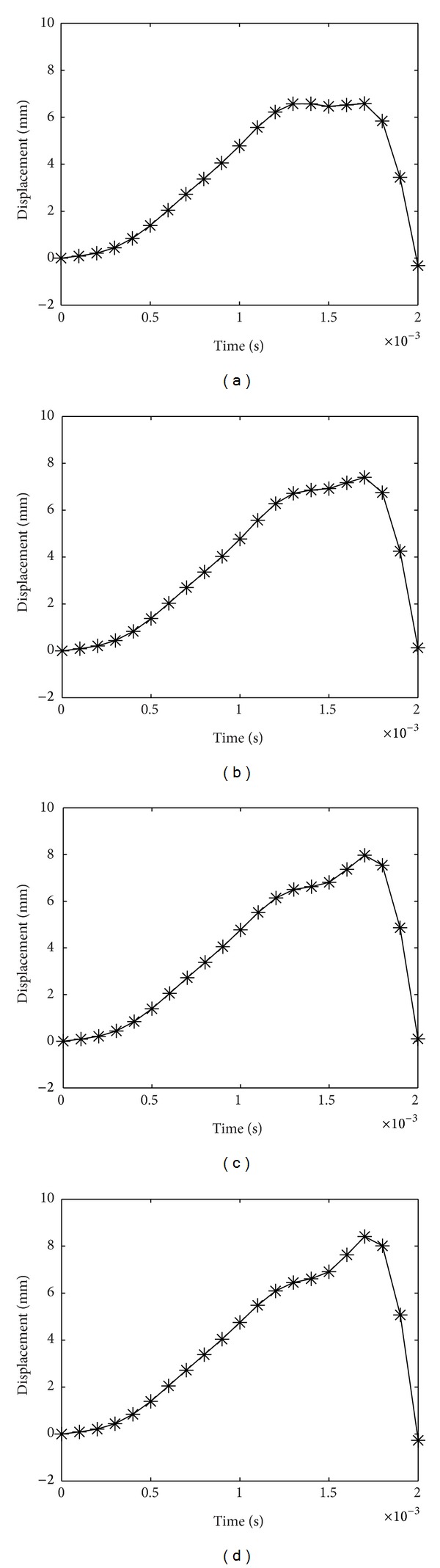
Displacement history of laminate with *A*_*r*_ = 0.63 for (a) [0/0], (b) [30/0], (c) [50/0], and (d) [90/0] laminates.

Figure 2 shows the displacement history of the plate with *A*_*r*_ = 0.25 for [0/0], [30/0], [50/0], and [90/0] orientations. The maximum central displacements of the plates are 3.9 mm, 4.21 mm, 4.31 mm, and 4.45 mm, respectively. As the level of delamination reaches *A*_*r*_ = 0.63, the maximum displacements are 6.58 mm, 7.4 mm, 7.97 mm, and 8.41 mm, for fiber orientations of [0/0], [30/0], [50/0], and [90/0], respectively, as shown in Figure 3.

**Figure 4:**
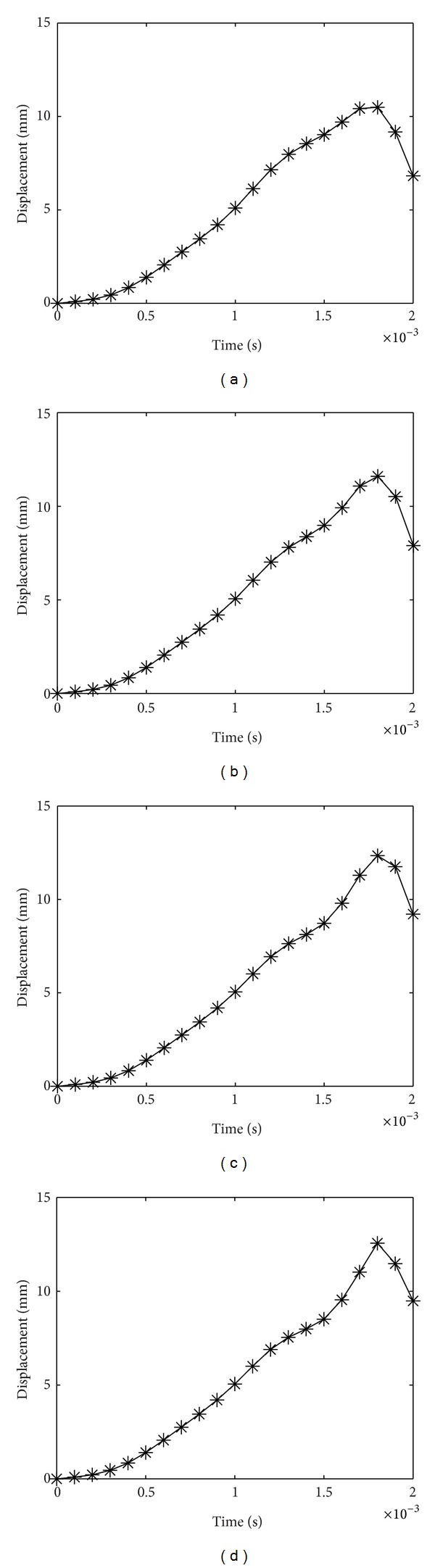
Displacement history of laminate with *A*_*r*_ = 0.88 for (a) [0/0], (b) [30/0], (c) [50/0], and (d) [90/0] laminates.

For the laminate with *A*_*r*_ = 0.88, the maximum central displacements are 10.45 mm, 11.6 mm, 12.34 mm, and 12.56 mm, for [0/0], [30/0], [50/0], and [90/0] laminates, respectively. The results are shown in Figure 4.

From Figures 2–4, it can be observed that as the top lamina fiber orientation increases, the central displacement increases as well. As the top and bottom fiber orientations increasingly differ, the two layers tend to act separately with a reduced global stiffness especially when delaminated area increases, resulting in a higher central displacement. As *A*_*r*_ rises, so does the central displacement. This phenomenon is well comprehended, since greater area of delamination causes degraded performance in laminates. Also, as *A*_*r*_ increases, the almost plateau displacement peak in the case of low *A*_*r*_ reduces to one pointed peak, and the displacement takes a longer time to subside. The negative values in some of the displacement graphs indicate that after the impact load vanishes, the plate rebounds and returns to its initial position.

### 3.2. Relationship between Area of Delamination and Maximum Central Displacement

Define the displacement ratio at any given time as follows:

(22) Displacement ratio (Dr) =maximum displacement of delaminated laminatemaximum displacement of perfectly bonded laminate.

**Figure 5:**
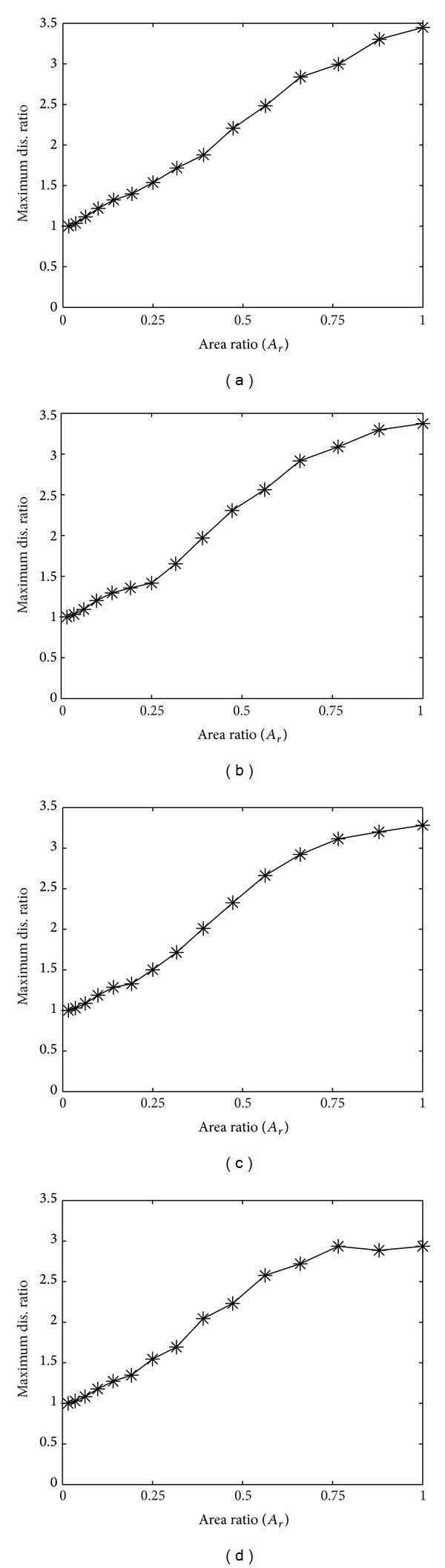
Relationship between area ratio and maximum displacement ratio for (a) [0/0], (b) [30/0], (c) [50/0], and (d) [90/0] laminates.

Figure 5 shows the relationship between the displacement ratios and the area ratios for [0/0], [30/0], [50/0], and [90/0] laminates, respectively. It is obvious that as *A*_*r*_ increases, so does *D*_*r*_. In other words, the maximum displacement is directly proportional to the area of delamination. It can also be observed that as *θ* of [*θ*/0] laminate increases, the maximum displacement decreases. Thus, the displacement is inversely proportional to the angle deviation of fiber orientation for two-layer laminate configuration. This implies that, when compared to its perfect condition, the higher the top and bottom lamina angles deviate, the lower the relative maximum displacement is.

### 3.3. Force-Displacement Relation of Various Laminates with Progressive Delamination

**Figure 6:**
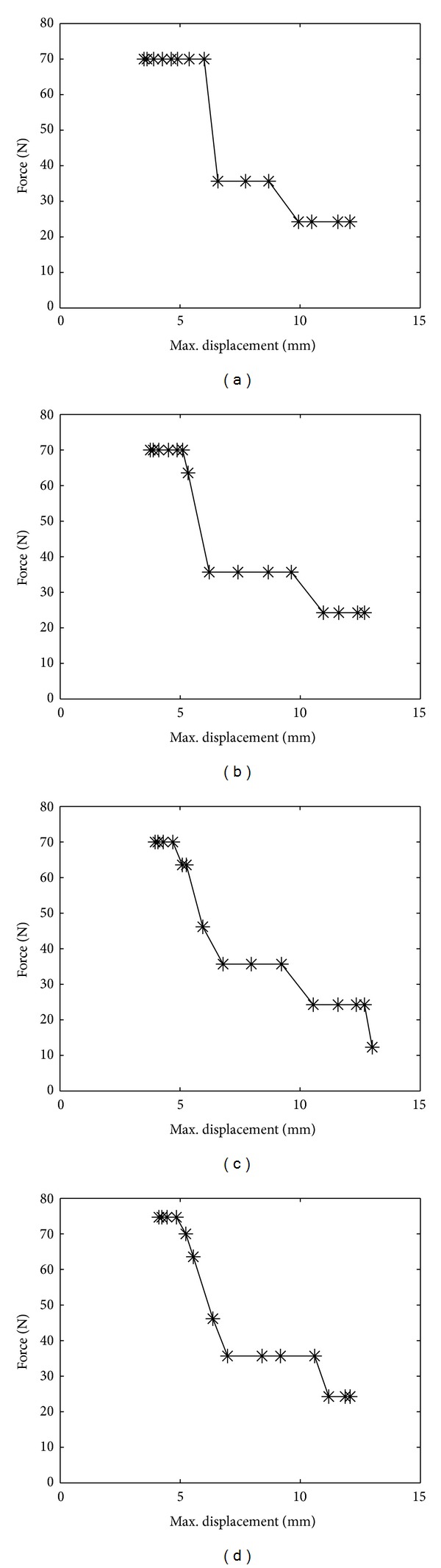
Force-displacement relationship of laminates with (a) [0/0], (b) [30/0], (c) [50/0], and (d) [90/0] orientations.

The delamination energy of each case can be established by calculating the area under the force-displacement curve, for each delamination case starting from *A*_*r*_ = 0.125 up to *A*_*r*_ = 1 as shown in Figure 6 for [0/0], [30/0], [50/0], and [90/0] laminates. Here, the maximum energies are 157.6 × 10^−3^ J, 177.7 × 10^−3^ J, 214.6 × 10^−3^ J, and 220.8 × 10^−3^ J, respectively.

It is evident that an increase in the top-bottom angle deviation leads to an increase in the absorbed energy. That means as *θ* in [*θ*/0] increases with the increase of delamination, the absorbed energy increases indicating that the plate experiences progressively higher intensity of damage. It is also noted that the force acts constantly at areas of low delamination. As the area of delamination increases, the force drops rapidly, particularly, when the plate's displacement reaches almost 6 mm for all cases. Then, several plateaus in force are observed as maximum displacement progresses. Accordingly, the displacement keeps increasing significantly with low magnitude of force, indicating that the plate is experiencing a severe damage.

## 4. Conclusion 

The paper presents finite element formation of a two-layer composite plate with localized interfacial delamination in the presence of impact load employing a well-defined virtually zero-thickness interface element. The following conclusions are obtained.

- As the area of delamination increases, the central displacement increases indicating that it is directly proportional to the area of delamination.
- Whenever the top fiber orientation *θ* in [*θ*/0] configuration increases, the central displacement increases.
- As *θ* in [*θ*/0] increases, the maximum central displacement ratio decreases. Thus, in relation to its corresponding perfect state, the maximum displacement of laminate is inversely proportional to the increase in *θ*.
- The increase in *θ* in [*θ*/0] leads to an increase in the absorbed energy, implying the increase of delamination and, hence, an enhanced damage state.

## Figures and Tables

**Table 1:** Plate's properties and dimensions.

Length and width of plate	100 mm × 100 mm
Stacking sequence	Upper lamina (0°) and lower lamina (45°)
Thickness of plate	0.5 mm
Material properties	*E*_1_ = 25 GPa, *E*_2_ = 1 GPa, *v*_12_ = 0.25, *v*_21_ = 0.25, *ρ* = 1 kg/m^3^, and *G*_12_ = 0.5 GPa

**Table 2:** Natural frequencies of the first eight mode shapes.

Mode sequence number	1	2	3	4	5	6	7	8
Shi et al. (2004) [4]	23.249	38.825	55.37	59.814	72.984	85.337	101.53	104.6
Present study	23	37	53	58	77	84	96	103
Difference, %	1.1	4.7	4.28	3	5.21	1.56	5.45	1.53
